# Correction: Photocatalytic [3 + 2]-annulation *via* sodium tetraarylborate: a fundamental approach for synthesizing 1,4,2-diazaborole analogs

**DOI:** 10.1039/d5sc90252e

**Published:** 2025-12-03

**Authors:** Hao-Ni Qin, Hao-Wen Jiang, Yi Zhao, Saira Qurban, Ke-Chun Wang, Peng-Fei Xu

**Affiliations:** a State Key Laboratory of Applied Organic Chemistry, College of Chemistry and Chemical Engineering, Lanzhou University Lanzhou 730000 Gansu China xupf@lzu.edu.cn; b Tecon Pharmaceutical Co., Ltd No. 109, Jintun Road Urumqi City 830023 Xinjiang China

## Abstract

Correction for ‘Photocatalytic [3 + 2]-annulation *via* sodium tetraarylborate: a fundamental approach for synthesizing 1,4,2-diazaborole analogs’ by Hao-Ni Qin *et al.*, *Chem. Sci.*, 2025, **16**, 2837–2842, https://doi.org/10.1039/D4SC08085H.

The authors regret that the structures of compounds **6a–6d** were incorrectly assigned in the original article. Single-crystal X-ray diffraction (SCXRD) analysis of **6a** has now confirmed the corrected structures. X-ray crystallographic data and CIF files of **6a** have been compiled in the Correction Supplementary Information available at https://doi.org/10.1039/D4SC08085H. The corrected Table 4 is shown below.

**Table 4:** Photocatalyzed [3 + 2]-annulation with styrenes

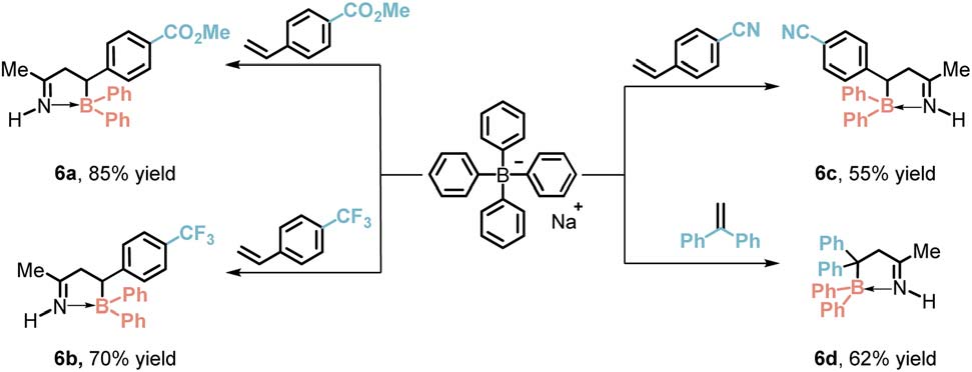

The authors take full responsibility for this error and sincerely apologise for any confusion or inconvenience it may have caused. They remain fully committed to maintaining the accuracy and integrity of their scientific work.

The Royal Society of Chemistry apologises for these errors and any consequent inconvenience to authors and readers.

